# variancePartition: interpreting drivers of variation in complex gene expression studies

**DOI:** 10.1186/s12859-016-1323-z

**Published:** 2016-11-25

**Authors:** Gabriel E. Hoffman, Eric E. Schadt

**Affiliations:** Department of Genetics and Genomic Sciences, Icahn Institute for Genomics and Multiscale Biology, Icahn School of Medicine at Mount Sinai, New York, USA

**Keywords:** Transcriptome profiling, RNA-seq, Linear mixed model

## Abstract

**Background:**

As large-scale studies of gene expression with multiple sources of biological and technical variation become widely adopted, characterizing these drivers of variation becomes essential to understanding disease biology and regulatory genetics.

**Results:**

We describe a statistical and visualization framework, variancePartition, to prioritize drivers of variation based on a genome-wide summary, and identify genes that deviate from the genome-wide trend. Using a linear mixed model, variancePartition quantifies variation in each expression trait attributable to differences in disease status, sex, cell or tissue type, ancestry, genetic background, experimental stimulus, or technical variables. Analysis of four large-scale transcriptome profiling datasets illustrates that variancePartition recovers striking patterns of biological and technical variation that are reproducible across multiple datasets.

**Conclusions:**

Our open source software, variancePartition, enables rapid interpretation of complex gene expression studies as well as other high-throughput genomics assays. variancePartition is available from Bioconductor: http://bioconductor.org/packages/variancePartition.

**Electronic supplementary material:**

The online version of this article (doi:10.1186/s12859-016-1323-z) contains supplementary material, which is available to authorized users.

## Background

High-throughput genomics assays have revolutionized our understanding of the molecular etiology of human disease, molecular biology of cell lineage and genetic regulation of gene expression. Transcriptome profiling in particular has been widely applied to detect variation in transcript levels attributable to differences in disease state, cell type or regulatory genetics. As transcriptome profiling studies have expanded in size and scope, they have grown increasingly complex and consider multiple sources of biological and technical variation. Recent studies have simultaneously considered multiple dimensions of variation to understand the impact of cell type [1], tissue type [2], brain region [3], experimental stimuli [4], time duration following stimulus [5] or ancestry [1, 4, 6] on the genetic regulation of gene expression. More studies are including a disease axis, for example to characterize the role of regulatory genetics on late onset Alzheimer’s disease in multiple brain regions [7].

The fundamental challenge in the analysis of complex datasets is to quantify and interpret the contribution of multiple sources of variation. Indeed the most pressing questions concern the relationship between these sources of variation. How does cell or tissue type affect the genetic regulation of gene expression, and does it vary by ancestry [1, 2]? What is the relative contribution of experimental stimulus versus regulatory genetics to variation in gene expression [5]? Is technical variability of RNA-seq low enough to study regulatory genetics and disease biology, and what are the major drivers of this technical variability [2, 8, 9]? A rich understanding of complex datasets requires answering these questions with both a genome-wide summary and gene-level resolution.

Standard computational workflows employ principal components analysis [10] and hierarchical clustering [11] to summarize genome-wide expression patterns, and differential expression [12–16] to perform gene-level analyses. Recently, statistical methods that decompose variation in gene expression into the variance attributable to multiple variables in the experimental design have yielded valuable insight into the biological and technical components driving expression variation [8, 17–22]. Moreover, linear mixed models have been widely used in the analysis and interpretation of genome-wide association studies [23–28].

The linear mixed model is uniquely suited to interpreting drivers of variation in complex gene expression studies. Yet the lack of a convenient workflow and scalable implementation for analysis and visualization have prevented wider application of this rich statistical framework. Applying this analysis framework to gene expression data currently requires particular expertise in regression modeling, computational statistics, the R programming language and data visualization. Even then, the time required to implement the analysis is often prohibitive.

As gene expression datasets become more complex, the analysis and interpretation of the data is becoming the rate-limiting step. We have developed the variancePartition software and workflow to facilitate rapid analysis and improve interpretation of complex gene expression datasets. The software and workflow enables any analyst to perform a sophisticated analysis and visualize the results in hours using a few lines of R code. variancePartition leverages the power of the linear mixed model [29–31] to jointly quantify the contribution of multiple sources of variation in high-throughput genomics studies. In applications to transcriptome profiling, variancePartition fits a linear mixed model for each gene and partitions the total variance into the fraction attributable to each aspect of the study design, plus the residual variation. Because it is built on the first principles of the linear mixed model, variancePartition has well characterized theoretical properties [29–31] and accurately estimates the variance fractions even for complex experimental designs where the standard ANOVA method is either inaccurate or not applicable. Moreover, variancePartition gives strong interpretations about the drivers of expression variation, and we demonstrate that these findings are reproducible across multiple datasets.

Here we apply variancePartition to four well-characterized gene expression studies to demonstrate how the workflow facilitates interpretation of drivers of expression variation in complex study designs with multiple dimensions of variation. We illustrate how variancePartition enables rapid interpretation of the drivers of expression variation in these complex datasets.

## Implementation

### Overview of the software

**Fig. 1:**
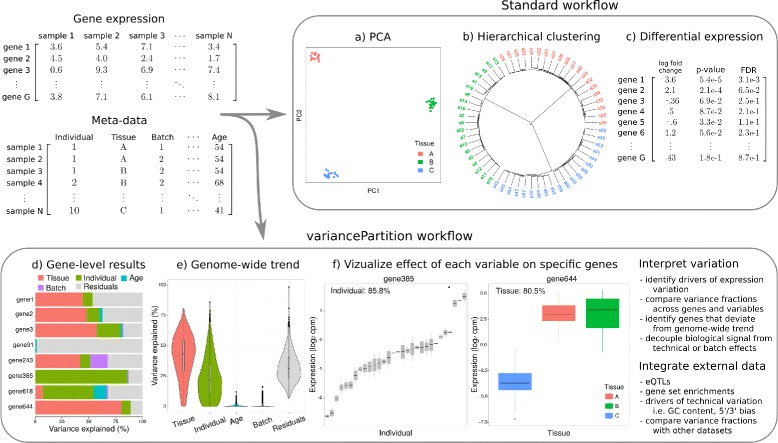
Analysis workflow of gene expression data and meta-data. Standard analysis consists of interpreting gene expression data with respect to variables in the metadata using genome-wide analysis such as **a** principal components analysis and **b** hierarchical clustering, and gene-level analysis such as **c** differential expression. The variancePartition workflow uses a rich statistical framework in the form of a linear mixed model and produces gene-level results and a genome-wide summary to simultaneously interpret gene expression data in the context of multiple variables in the metadata. The workflow produces **d** gene-level results quantifying the contribution of each metadata variable to the variation in expression of each gene, and **e** a violin plot to summarize the genome-wide trend and rank the total contribution of each variable. **f** The gene-level results can be used to identify genes that show high expression variation across individuals (i.e. gene385) or tissue (i.e. gene644). Furthermore, variancePartition facilitates examination of specific genes, and integrating external data enables further interpretation of the drivers of expression variation

The variancePartition R package implements a computational workflow (Fig. 1) that is complementary to standard analyses and provides particular insight into datasets with multiple dimensions of variation. variancePartition provides a user-friendly, parallelized interface for genome-wide analysis and publication quality visualizations to examine the results. Because the variance fractions are simple to describe and interpret, variancePartition can give particular insight into how each dimension of variation contributes to transcriptional variability. A typical variancePartition analysis comprises: 1) fitting a linear mixed model to quantify the contribution of each dimension of variation to each gene, 2) visualizing the results, and 3) integrating additional data about each gene to interpret drivers of this variation. The variancePartition workflow requires only a few lines of R code for pre-processing, analysis and visualization and this enables rapid interpretation of complex datasets.

The variancePartition software is implemented in R and is optimized for genome-wide analysis of large-scale transcriptome profiling datasets. variancePartition uses the packages lme4 [29] foreach [32], iterators [33] and doParallel [34] to efficiently fit a linear mixed model for each gene in parallel on a multicore machine with a small memory footprint. The precision weights from limma/voom [15] are seamlessly incorporated into the analysis workflow. Built-in publication quality visualizations are implemented in ggplot2 [35]. The variancePartition software including extensive documentation is available from http://bioconductor.org/packages/variancePartition and is compatible with Bioconductor ≥v3.2 for R ≥v3.2.

### Linear mixed model framework

variancePartition summarizes the contribution of each variable in terms of the fraction of variation explained (FVE). While the concept of FVE is widely applied to univariate regression by reporting the *R* ^2^ value from a simple linear model, variancePartition extends FVE to applications with complex study designs with multiple variables of interest. The linear mixed model framework of variancePartition allows multiple dimensions of variation to be considered jointly in a single model and accommodates discrete variables with a large number of categories. This analysis has a similar motivation as the standard ANOVA method. Yet the linear mixed model framework has several statistical and practical advantages that make it more accurate and generally applicable to complex study designs with multiple dimensions of variation (Additional file 1).

Each gene is analyzed separately using the linear mixed model [29–31]

1 $$\begin{array}{@{}rcl@{}} y &=& \sum_{j} X_{j}\beta_{j} + \sum_{k} Z_{k} \alpha_{k} + \varepsilon  \end{array} $$

2 $$\begin{array}{@{}rcl@{}} \alpha_{k} &\sim& \mathcal{N}(0, \sigma^{2}_{\alpha_{k}}) \end{array} $$

3 $$\begin{array}{@{}rcl@{}} \varepsilon &\sim& \mathcal{N}(0, \sigma^{2}_{\varepsilon}) \end{array} $$

where *y* is the expression of a single gene across all samples, *X*_*j*_ is the matrix of *j* ^*th*^ fixed effect with coefficients *β*_*j*_,*Z*_*k*_ is the matrix corresponding to the *k* ^*th*^ random effect with coefficients *α*_*k*_ drawn from a normal distribution with variance $\sigma ^{2}_{\alpha _{k}}$. The noise term, *ε*, is drawn from a normal distribution with variance $\sigma ^{2}_{\varepsilon }$. All parameters are estimated with maximum likelihood [29] as simulations under a range of experimental designs indicate that this approach gives the most accurate FVE estimates (Additional file 1: Figures S1–S4).

Variance terms for the fixed effects are computed using the *post hoc* calculation

4 $$\begin{array}{@{}rcl@{}} \hat{\sigma}^{2}_{\beta_{j}} = var(X_{j} \hat{\beta}_{j}). \end{array} $$

The total variance is

5 $$\begin{array}{@{}rcl@{}} \hat{\sigma}^{2}_{Total} = \sum_{j} \hat{\sigma}^{2}_{\beta_{j}} + \sum_{k} \hat{\sigma}^{2}_{\alpha_{k}} + \hat{\sigma}^{2}_{\varepsilon} \end{array} $$

so that the fraction of variance explained by the *j* ^*th*^ fixed effect is

6 $$\begin{array}{@{}rcl@{}} \hat{\sigma}^{2}_{\beta_{j}} /\hat{\sigma}^{2}_{Total}, \end{array} $$

by the *k* ^*th*^ random effect is

7 $$\begin{array}{@{}rcl@{}} \hat{\sigma}^{2}_{\alpha_{k}} /\hat{\sigma}^{2}_{Total}, \end{array} $$

and the residual variance is

8 $$\begin{array}{@{}rcl@{}} \hat{\sigma}^{2}_{\varepsilon} /\hat{\sigma}^{2}_{Total}.  \end{array} $$

In the standard application of variancePartition, these fractions sum to 1 and are always positive by definition. Moreover, the fraction of variation is also interpretable in terms of intra-class correlation, a metric used to assess biological and technical reproducibility [31, 36]. Each gene is processed separately so that only visualization and reporting of genome-wide summary statistics use the results from multiple genes.

### Parameter estimation

The formulation of the linear mixed model is very general and includes as special cases models where only fixed effects or only random effects are used. When only fixed effects are used, this model corresponds to a fixed effects analysis of variance (ANOVA) where parameters can be estimated with ordinary least squares. When random effects are specified, the variance terms can be estimated with maximum likelihood or restricted maximum likelihood (REML) [37]. Since REML does not directly estimate parameters for fixed effects, these coefficients are estimated after the fact by plugging in estimates for the variance components [29].

We focus on the most general case (i.e. mixed models) that includes both fixed and random effects. In this case parameters can be estimated with maximum likelihood. Maximum likelihood estimates are used exclusively in the main text and are the default in the variancePartition software when random effects are specified because this method performs best in simulations.

### Relationship to existing methods

The fixed effects ANOVA model has been widely applied for decades to decompose variance into multiple components of variation [38]. Yet this approach is often inadequate to address the questions that are posed by complex gene expression datasets.

The linear mixed model used by variancePartition has three distinct advantages compared to ANOVA. First, by placing a Gaussian prior on variables modeled as random effects, the linear mixed model more accurately estimates the fraction of variance explained. Even as the number of categories in a discrete variable increases, the linear mixed model still produces accurate estimates because the prior shrinks the estimate for each category towards the zero. Conversely, the fixed effects ANOVA is fit with a linear regression model using ordinary least squares. This method is known to suffer from overfitting and over-estimates the variance fractions for variables with many categories. These properties are well established [31, 38, 39] and are consistent with our simulation study (Additional file 1).

Second, the linear mixed model can decompose variance into multiple components in situations where the fixed effects ANOVA cannot be applied because the design matrix is degenerate (i.e. singular). This situation is very common for the types of question of relevant to complex gene expression studies. For example, sex and ancestry are invariant properties of an individual, so jointly analyzing variation across these 3 dimensions of variation involves a degenerate design matrix. In cases like these, the linear mixed model can accurately estimate the desired variance fractions (Additional file 1), while ANOVA will fail to estimate any of these values because the parameters are not identifiable. Thus ANOVA is inadequate for the type of analysis we performed here with variancePartition using linear mixed model.

Finally, the linear mixed model can quantify how variation attributable to one aspect of the study design depends on another, such as the case of cross-individual expression variation depending on tissue/cell type. ANOVA does not have this capability.

### Interpretation of percent variance explained

The percent variance explained can be interpreted as the intra-class correlation (ICC). Consider the simplest example of the *i* ^*th*^ sample from the *k* ^*th*^ individual

9 $$\begin{array}{@{}rcl@{}} y_{i,k} &=& \mu + \alpha_{k} + \varepsilon_{i,k} \end{array} $$

10 $$\begin{array}{@{}rcl@{}} \alpha_{k} &\sim& \mathcal{N}(0, \sigma^{2}_{\alpha}) \end{array} $$

11 $$\begin{array}{@{}rcl@{}} \varepsilon_{i,k} &\sim& \mathcal{N}(0, \sigma^{2}_{\varepsilon})  \end{array} $$

with only an intercept term, one random effect corresponding to individual, and an error term. In this case ICC corresponds to the correlation between two samples from the same individual. This value is equal to the fraction of variance explained by individual. For example, consider the correlation between samples from the same individual:

12 $$\begin{array}{@{}rcl@{}} \text{ICC} &=& cor(y_{1,k}, y_{2,k}) \end{array} $$

13 $$\begin{array}{@{}rcl@{}} &=& cor(\mu + \alpha_{k} + \varepsilon_{1,k}, \mu + \alpha_{k} + \varepsilon_{2,k}) \end{array} $$

14 $$\begin{array}{@{}rcl@{}} &=& \frac{cov(\mu + \alpha_{k} + \varepsilon_{1,k}, \mu + \alpha_{k} + \varepsilon_{2,k})}{ \sqrt{var(\mu + \alpha_{k} + \varepsilon_{1,k}) var(\mu + \alpha_{k} + \varepsilon_{2,k})}} \end{array} $$

15 $$\begin{array}{@{}rcl@{}} &=& \frac{cov(\alpha_{k}, \alpha_{k})}{\sigma^{2}_{\alpha} + \sigma^{2}_{\varepsilon}} \end{array} $$

16 $$\begin{array}{@{}rcl@{}} &=& \frac{\sigma^{2}_{\alpha}}{\sigma^{2}_{\alpha} + \sigma^{2}_{\varepsilon}}  \end{array} $$

The correlation between samples from different individuals is:

17 $$\begin{array}{@{}rcl@{}} &=& cor(y_{1,1}, y_{1,2}) \end{array} $$

18 $$\begin{array}{@{}rcl@{}} &=& cor(\mu + \alpha_{1} + \varepsilon_{1,1}, \mu + \alpha_{2} + \varepsilon_{1,2}) \end{array} $$

19 $$\begin{array}{@{}rcl@{}} &=& \frac{cov(\alpha_{1}, \alpha_{2})}{\sigma^{2}_{\alpha} + \sigma^{2}_{\varepsilon}} \end{array} $$

20 $$\begin{array}{@{}rcl@{}} &=& \frac{0}{\sigma^{2}_{\alpha} + \sigma^{2}_{\varepsilon}} \end{array} $$

21 $$\begin{array}{@{}rcl@{}} &=& 0  \end{array} $$

This interpretation in terms of fraction of variation explained (FVE) naturally generalizes to multiple variance components [31]. See Additional file 1 for more details.

### Variation across individual within subsets of the data

The linear mixed model underlying variancePartition allows the effect of one variable to depend on the value of another variable. Statistically, this is called a varying coefficient model [31]. This analysis examines the expression variation across individuals within multiple cell types, or another subset of the data. A given sample is only from one cell type, so this analysis asks a question about a subset of the data. The data is implicitly divided into subsets based on cell type and variation explained by individual is evaluated within each subset. This subsetting means that the variance fractions no longer sum to 1, but the model still allows ranking of dimensions of variation based on genome-wide contribution to variance and enables analysis of gene-level results. See the Additional file 1 for more details.

### Modeling measurement error in RNA-seq data

Uncertainty in the measurement of RNA-seq data can be modeled with observation-level precision weights that model the relationship between expression magnitude and sampling variance [15]. variancePartition naturally incorporates these precision weights to create a heteroskedastic linear mixed model [29] that can explicitly account from the measurement uncertainty due to the finite count nature of RNA-seq data.

Let the precision *w*_*i*_ denote the inverse of the variance of the observation *y*_*i*_ for the *i* ^*th*^ observation. The precisions can be used to re-weight the samples in a regression to account for the variation in the uncertainty about each observation. Weighting by the precision upweights samples with low measurement error and down weights samples with high measurement error. Denoting the vector of precision weights for a single gene across all samples as *w*, the model is fit by weighting the residual variance from equation (8)

22 $$\begin{array}{@{}rcl@{}} \varepsilon &\sim& \mathcal{N}(0, \text{diag}(w)\sigma^{2}_{\varepsilon}). \end{array} $$

These weights are estimated using limma/voom [15] in a preprocessing step and are then incorporated into the variancePartition analysis.

## Results

### Analysis of GEUVADIS RNA-seq dataset

Consider 660 RNA-seq experiments from the GEUVADIS study [6, 8] of lymphoblastoid cell lines from 462 individuals of 5 ancestries and 2 sexes sequenced across 7 laboratories. For a single gene, the total variance can be partitioned into the contributions of these components of variation plus residual variance:

23 $$\begin{array}{@{}rcl@{}} \sigma^{2}_{Total} = \sigma^{2}_{Individual} + \sigma^{2}_{Lab} + \sigma^{2}_{Ancestry} + \sigma^{2}_{Sex} + \sigma^{2}_{\varepsilon}. \end{array} $$

The contribution of each driver of variation can be interpreted based on the fraction of total variation it explains. Thus the fraction of variance due to variation across individuals is

24 $$\begin{array}{@{}rcl@{}} \sigma^{2}_{Individual}/\sigma^{2}_{Total}, \end{array} $$

and the fractions from all components of variation sum to 1.

**Fig. 2:**
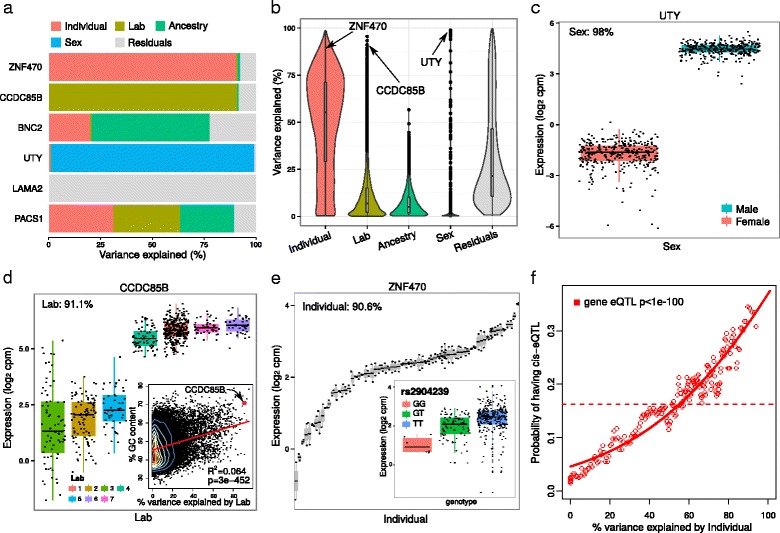
Analysis of GEUVADIS dataset identifies drivers of expression variation. **a** Total variance for each gene is partitioned into the fraction attributable to each dimension of variation in the study. **b** Violin and box plots of percent variation in gene expression explained by each variable. Three representative genes and their major sources of variation are indicated. **c** Boxplot of UTY expression stratified by sex. **d** Boxplot of CCDC85B expression stratified by lab. Inset shows scatter plot of percent GC content versus percent variance explained by lab. Red line indicates linear regression line with coefficient of determination and *p*-value shown. **e** Boxplot of ZNF470 expression stratified by individual for a subset of individuals with at least 1 technical replicate. Inset illustrates a cis-eQTL for ZNF470 where expression is stratified by genotype at rs2904239. **f** Probability of each gene having a cis-eQTL plotted against the percent variance explained by individual. Dashed lines indicate the genome-wide average probability (i.e. 18% of genes have a detected eQTL in this dataset), and curves indicate logistic regression smoothed probabilities as a function of the percent variance explained by individual. Points indicate a sliding window average of the probability of genes in each window having a cis-eQTL. Window size is 200 genes with an overlap of 100 genes between windows. The *p*-value indicates the probability that a more extreme coefficient relating the eQTL probability to percent variation explained by individual is observed under the null hypothesis

Applying variancePartition to the GEUVADIS [6, 8] dataset illustrates how the method can decouple biological and technical variation, and further decompose biological variation into multiple components. Expression variation across individuals, ancestries and sexes is biological, variation across the labs where the samples were sequenced comprise technical artifacts, while the residual variation remains uncharacterized. Results from representative genes illustrate how variancePartition identifies genes where the majority of variation in expression is explained by a single variable such as individual or sex, while variation in other genes is driven by multiple variables (Fig. 2 a). Since the variance fractions sum to 1 for each gene, it is simple to compare results across genes and across sources of variation. Visualizing these results genome-wide illustrates that variation across individuals is the major source of expression variation and explains a median of 55.1% of variance genome-wide (Fig. 2 b). The median variance explained by laboratory (6.8%), ancestry (4.9%) and sex (0%) is substantially smaller. We note that the variance explained by individual increases to 63.8% when ancestry is removed from the analysis since ancestry is a biological property of each individual (Additional file 1: Figure S5).

Yet particular genes show substantial deviation from the genome-wide trend. This is particularly noticeable for sex, where of the 51 genes for which sex explains more that 10% variance 46 are on the X or Y chromosomes. For example, variation across sex explains 98% variance in UTY on the Y chromosome (Fig. 2 c). While differential expression measures the differences in mean expression between the sexes, variancePartition measures the variance within and between each sex. This analysis indicates that variation across sexes explains very little variation genome-wide, but has a strong effect on a small number of genes.

Integrating additional data with gene-level results from variancePartition can give a clear interpretation of the drivers of variation. For example, 91.1% of variation in CCDC85B is explained by variation across laboratory. This gene has a very high GC content of 70.9% and is consistent with the genome-wide pattern where the degree of variation across laboratories is positively correlated with GC content (Fig. 2 d). While technical variation in RNA-seq is known to depend on GC content [8, 9], variancePartition gives a clear illustration of how the effect of technical artifacts varies substantially across genes. Moreover, this analysis can be used to identify other correlates underlying technical issues in expression variation.

In addition, variancePartition gives a strong interpretation to genes whose expression varies across individuals by relating the gene-level results to cis-regulatory variation. For example, the fact that 90.6% of variation in ZNF470 is explained by individual suggests that this variation is driven by genetics, and, in fact, ZNF470 has a cis-eQTL (Fig. 2 e). This observation is also seen genome-wide, as genes with a greater fraction of variation across individuals have a significantly higher probability of having a cis-eQTL detected in this study (Fig. 2 f). This analysis explicitly demonstrates how expression variation across individuals is driven by cis-regulatory variation.

### Analysis of SEQC RNA-seq dataset

The Sequencing Quality Control (SEQC) project [9] evaluated the technical reproducibility of RNA-seq data by sequencing the same 4 RNA samples at 6 laboratories, using 108 total library constructions and up to 8 lanes on each of 11 Illumina HiSeq 2000 flowcells for a total of 1580 RNA-seq experiments. The goal of the study was to determine the degree to which these technical factors explain variation in gene expression measurements. This complex dataset has multiple levels of variation and variancePartition provides a rigorous statistical framework to quantify and interpret these sources of variation in a single analysis.

**Fig. 3:**
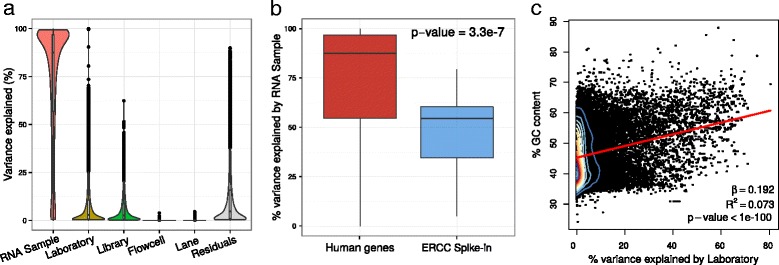
Analysis of Sequencing Quality Control (SEQC) dataset decouples sources of technical variation. **a** Violin and box plots of percent variation in gene expression explained by each variable. **b** Boxplot of percent variance explained by RNA sample for human genes and External RNA Controls Consortium (ERCC) spike-in controls. *P*-value is from one-sided Mann-Whitney test. **c** Scatter plot of percent GC content and percent variance explained by laboratory. Red line indicates linear regression line with regression coefficient, coefficient of determination and *p*-value shown

As expected, variation across the 4 RNA samples is the major axis of variation, explaining a median of 87.5% of variation in expression (Fig. 3 a). But the real interest is in the sources of technical variability. The fact that the technical variables laboratory (2.93%), library (2.55%), flowcell (0.0057%), and lane (0.0000000038%) explain a small fraction of the total variation indicate that these RNA-seq experiments were highly reproducible genome-wide. Interpreting these values in terms of the intra-class correlation indicates that two experiments from the sample RNA sample but which differ in all other aspects of the study design are highly correlated (median 87.5%). Conversely, two experiments from the same lane, but different RNA samples, etc, show negligible correlation as is expected when technical variation is low.

Analysis and visualization with variancePartition succinctly illustrates that while variation across laboratories and library constructions is not negligible, it is small compared with the magnitude of biological variation for the large majority of genes. Moreover, variation across flowcells and lanes is very small in this dataset. Thus variancePartition illustrates that RNA-seq data is highly reproducible genome-wide with a small subset of genes showing large technical artifacts.

However, there are notable deviations from these genome-wide trends. First, there are a set of transcripts that show little variation between the 4 RNA samples and, in fact, these correspond to spike-in synthetic RNA added to each sample at a standardized concentration to act as controls having equal abundance in all experiments [40]. As expected, spike-in transcripts show significantly less variation across the 4 RNA samples than human genes (Fig. 3 b). Second, although technical effects are low for most genes, a small number of genes show high variation across laboratories and library constructions. In fact, the fraction of variation across laboratories correlates with the GC content of each gene (Fig. 3 c), and recapitulates the known role of GC content with reproducibility of RNA-seq data [8, 41–43].

### Analysis of ImmVar microarray dataset

**Fig. 4:**
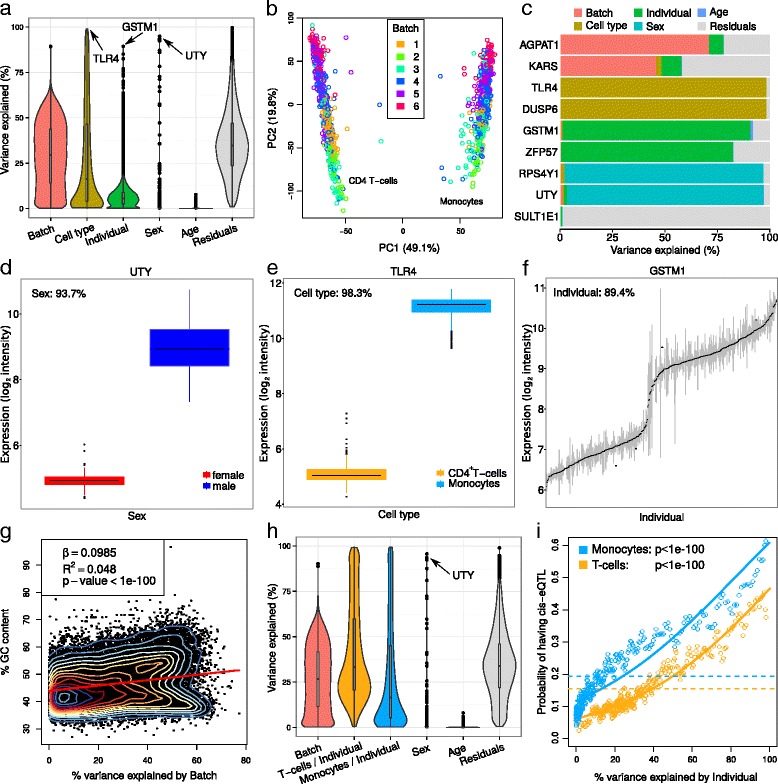
Analysis of ImmVar dataset interprets multiple dimensions of expression variation. **a** Violin and box plots of percent variation in gene expression explained by each variable. **b** Principal components analysis of gene expression with experiments colored by batch. **c** Total variance for each gene is partitioned into the fraction attributable to each dimension of variation in the study design. **d** Expression of UTY stratified by sex. **e** Expression of TLR4 stratified by cell type. **f** Expression of GSTM1 stratified by individual. **g** Scatter plot of percent GC content and percent variance explained by batch. Red line indicates linear regression line with regression coefficient, coefficient of determination and *p*-value shown. **h** Results from variancePartition analysis allowing the contribution of individual to vary in each cell type. **i** Probability of each gene having a cis-eQTL plotted against the percent variance explained by individual within each cell type. Dashed lines indicate the genome-wide average probability, and curves indicate logistic regression smoothed probabilities as a function of the percent variance explained by individual within each cell type. Points indicate a sliding window average of the probability of genes in each window having a cis-eQTL. Window size is 200 genes with an overlap of 100 genes between windows. The *p*-value indicates the probability that a more extreme coefficient relating the eQTL probability to percent variation explained by individual is observed under the null hypothesis

The Immune Variation (ImmVar) project assayed gene expression in CD14 ^+^CD16 ^−^ monocytes and CD4 ^+^ T-cells on the Affymetrix Human Gene 1.0 ST Array platform in order to characterize the role of cell type in genetic regulation of gene expression [1]. Analysis of 398 individuals with data from both cell types reveals that multiple variables contribute to expression variation in this dataset (Fig. 4 a). Since variancePartition reports the contribution of each variable while simultaneously correcting for all other values, it is apparent that the variation across cell types is the strongest biological driver of variation (16.4%) followed by variation across individuals (5.6%). Although cell type has a smaller median effect than batch, it is notable that cell type explains >50*%* of the variation for 4,591 genes. The observation that batch and cell type are the strongest drivers of variation is largely consistent with results from principal components analysis (PCA) (Fig. 4 b). We note that the relationship between variancePartition and PCA depends on both the fraction of expression variation explained by a particular variable across all genes as well as the dimension of the variable. While variation across the 2 cell types explains less expression variation than variation across the 6 batches, the first principal component separates samples by cell type because this variable spans a lower-dimensional space.

Meanwhile, sex drives expression variation in a small number of genes, while the age of each individual has a negligible effect. We note that despite the large batch effect observed in this dataset, the biological variation across cell type, individual and sex are still large enough to make meaningful conclusions about cell-specific regulatory genetics when this technical effect is accounted for [1].

Moreover, variancePartition identifies genes that vary along different aspects of the study design (Fig. 4 c), and visualization of a subset of these genes illustrates the strong expression differences when stratified by sex, cell type and individual (Fig. 4 d–f). variancePartition enables further interpretation of the batch effect because it gives results at a gene-level resolution. The samples were processed in 6 technical batches and this axis of variation explains a median of 29.4% of total variation, indicating a large technical effect. Consistent with other analyses, the fraction of variation explained by batch at the gene-level is positively correlated with GC content (Fig. 4 g).

By leveraging the flexibility of the linear mixed model, variancePartition can quantify the variation across individuals within each cell type. Since the variance is analyzed within multiple subsets of the data and each sample is only in a single subset, the total variation explained no longer sums to 1 as it does for standard application of variancePartition. Yet the results allow ranking of dimensions of variation based on genome-wide contribution to variance and enables analysis of gene-level results (Additional file 1). This analysis uses the fact that 34 individuals within monocytes have at least 1 technical replicate, while 41 individuals within T-cells have at least 1 technical replicate.

The variation across individuals within T-cells (median 33.2%) and monocytes (median 16.4%) is substantially larger than when the two cell types were combined (Fig. 4 h). The fact that the contribution of individual varies between cell types is consistent with cell-specific regulatory genetics [1]. Finally, the fraction of variation explained by individual within each cell type at the gene-level is directly related to the probability of each gene having cis-eQTL within the corresponding cell type (Fig. 4 i).

### Analysis of GTEx RNA-seq dataset

**Fig. 5:**
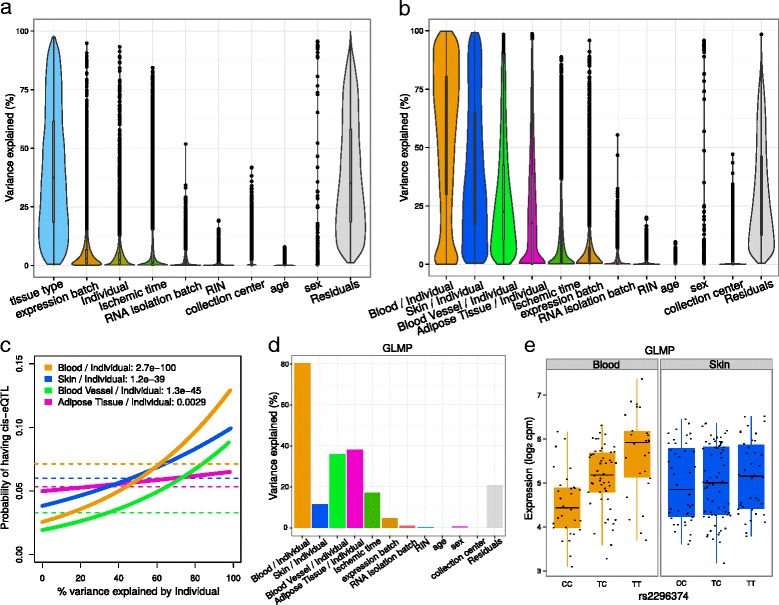
Analysis of GTEx dataset identifies drivers of expression variation at multiple levels. **a** Violin and box plots of percent variation in gene expression explained by each variable. **b** Results from variancePartition analysis allowing the contribution of individual to vary in each tissue. **c** Probability of each gene having a cis-eQTL plotted against the percent variance explained by individual within each tissue. Dashed lines indicate the genome-wide average probability, and curves indicate logistic regression smoothed probabilities as a function of the percent variance explained by individual within each tissue. The *p*-value indicates the probability that a more extreme coefficient relating the eQTL probability to percent variation explained by individual is observed under the null hypothesis. **d** Fraction of variation in GLMP explained by each source of variation. **e** GLMP has a cis-eQTL active in blood but not skin

Application of variancePartition to *post mortem* RNA-seq data of multiple tissues tissues from the GTEx Consortium [2] decouples the influence of multiple biological and technical drivers of expression variation. We analyzed 489 experiments from 103 individuals in 4 tissues (blood, blood vessel, skin and adipose tissue) in order to restrict the analysis to tissues with RNA-seq data for most individuals (Additional file 1: Table S1). Variation across tissues is the major source of variation (median 37.4%) while the technical variables expression batch (2.9%), ischemic time (1.2%), RNA isolation batch (0.4%), and RIN (0.2%) have a moderate effect on expression variation genome-wide (Fig. 5 a). Variation across expression batches is correlated with GC content but to a lesser degree that other datasets (Additional file 1: Figure S6). Cumulatively, these technical variables explain only 4.7% of the total expression variation. Concerns about reliability of RNA-seq data from *post mortem* samples has been raised due to the potential effects of RNA degradation following cell death [44, 45]. variancePartition analysis indicates that variation in ischemic time has as relatively small effect genome-wide and the fraction of variance it explains is comparable to technical effects, yet the effect varies substantially across genes.

The flexibility of the linear mixed model framework allows variancePartition to analyze cross-individual variation within each tissue. We note again that since the variance is analyzed within multiple subsets of the data, the total variation explained no longer sums to 1 here. While variation across individuals explains only a median of 2.3% of variation when all tissues types are considered together, there is substantial variation across individuals within each tissue separately (Fig. 5 b). Cross-individual variation is highest in blood (median 60.3%), while skin (36.5%), blood vessel (22.5%), and adipose tissue (17.7%) exhibit lower cross-individual variation. The fraction of variation explained by individual within each tissue is directly related to the probability of each gene having a cis-eQTL within the corresponding tissue (Fig. 5 c). This association is not as strong as in other datasets likely due to the smaller number of individuals and to the relatively small fraction of variation across individuals in adipose tissue.

At the gene-level, variancePartition can prioritize genes based on multiple criteria. For examples, GLMP exhibits higher variation across individuals within blood but low variation in skin (Fig. 5 d). This is consistent of a tissue-specific regulatory variation, and, in fact, the cis-eQTL rs2296374 influences gene expression in blood but not in skin (Fig. 5 e).

## Discussion

As the scope of gene expression studies continues to expand, the need to quantify and interpret multiple drivers of expression variation is becoming essential. Here we present variancePartition, a publicly available software package that leverages the power of the linear mixed model to quantify the contribution of multiple sources of variation in complex gene expression datasets. For each gene, this analysis partitions the total expression variance into the fraction attributable to each aspect of the study design. A variancePartition analysis gives a genome-wide summary of the drivers of variation, but also produces gene-level results to identify genes that deviate from the genome-wide trend.

The fraction of expression variation is easily interpretable across genes, drivers of variation and datasets. Thus variancePartition produces a more detailed and quantitative genome-wide overview than the standard principal components analysis (PCA) [10] and hierarchical clustering (HC) [11] approaches. PCA and HC focus on the major axis of variation, and they overlook the secondary drivers of variation that can be well characterized with variancePartition. Moreover, the gene-level results from variancePartition indicate genes that deviate from the genome-wide trend and integration with additional data can enable a further interpretation. While PCA and HC do not give gene-level results, differential expression (DE) analysis reports gene-level fold change and corresponding *p*-value for each aspect of the study design. Yet DE analysis does not produce a clear genome-wide summary, and the fold change and *p*-values are not easily comparable across multiple drivers of variation.

Analysis of publicly available gene expression studies demonstrate that variancePartition recovers striking patterns of biological and technical variation that are reproducible across multiple datasets. At a genome-wide level, expression variation across individuals and cell types is large enough to overcome the technical variation of transcriptome profiling. Yet at the gene-level there is substantial deviation from the genome-wide trend due to a range of biological and technical factors. By quantifying the variance attributable to each aspect of the study design, variancePartition facilitates the interpretation of these gene-level effects in the context of additional information. We demonstrate reproducible findings that cross-individual variation is driven by cis-eQTL’s and technical variation across laboratories associated with GC content. Moreover, variation across individuals and the relationship to cis-eQTL’s depend on the cell or tissue type.

## Conclusions

The variancePartition workflow and implementation makes the rich linear mixed model framework easily applicable for interpreting drivers of variation in complex gene expression data. variancePartition provides a general statistical and visualization framework for studying drivers of variation in RNA-seq datasets in many types of high-throughput genomic assays including RNA-seq (gene-, exon- and isoform-level quantification, splicing efficiency), protein quantification, metabolite quantification, metagenomic assays, methylation arrays and epigenomic sequencing assays. Although we have focused here on large-scale studies, variancePartition analysis has given valuable insight into RNA-seq datasets with as few as 20 samples. The variancePartition software is an open source R package and is freely available on Bioconductor. The software can easily be applied to RNA-seq quantifications from featureCounts [46], HTSeq [47], kallisto [48], sailfish [49], salmon [50], RSEM [51] and cufflinks [52] which have been processed in R with limma/voom [15], DESeq2 [16], tximport [53] and ballgown [54]. The software provides a user-friendly interface for analysis and visualization with extensive documentation, and will enable routine application to a range of genomics datasets.

## Availability and requirements

- **Project name:** variancePartition
- **Project home page:** http://bioconductor.org/packages/variancePartition
- **Operating systems:** Linux, Mac OS X, Windows
- **Programming language:** R ≥ v3.2
- **Other requirements:** Bioconductor ≥ v3.2
- **License:** GPL-2

## Additional file

Additional file 1 Supplementary Note. Additional details describing statistical methods and software. Includes results from simulation study and additional figures from data analysis. (PDF 727 kb)

## Abbreviations

ANOVA: Analysis of variance
DE: Differential expression
eQTL: Expression quantitative trait locus
FVE: Fraction of variance explained
HC: Hierarchical clustering
ICC: Intra-class correlation
IQR: Inter-quartile range
PCA: Principal components analysis
REML: Restricted maximum likelihood
