# Supplementary material for: variancePartition: interpreting drivers of variation in complex gene expression studies
Source: BMC Bioinformatics. 2016 Nov 25;17:483. doi: 10.1186/s12859-016-1323-z (PMC5123296; doi:10.1186/s12859-016-1323-z)
Supplement: Additional file 1 — Supplementary Note. Additional details describing statistical methods and software. Includes results from simulation study and additional figures from data analysis. (PDF 727 kb) [file 12859_2016_1323_MOESM1_ESM.pdf]

Supplementary Note

variancePartition: Interpreting drivers of variation in complex gene expression studies

Gabriel E. Hoffman[1,2*] and Eric E. Schadt[1,2]

[1]Icahn Institute for Genomics and Multiscale Biology
[2]Department of Genetics and Genomic Sciences
Icahn School of Medicine at Mount Sinai, New York, NY, USA
*Corresponding authors: gabriel.hoffman@mssm.edu

Contents

1 Supplementary Note **2**
1.1 Relation to previous work 2
1.2 Interpretation of percent variance explained 2
1.3 Variation within multiple subsets of the data 4
1.4 Software 5
1.5 Simulations 6
1.6 Processing expression data 8

2 Supplementary Figures **10**

3 Supplementary Tables **16**

1 Supplementary Note

1.1 Relation to previous work

Statistical methods to apportion variance to multiple experimental variables have recently yielded valuable insight into complex gene expression datasets:

- 't Hoen, et al. [1] used a fixed effects ANOVA model in R to examine the contribution of individual and laboratory in the GEUVADIS RNA-seq data, and examined the effect of technical factors to expression variability in a second analysis.
- Melé, et al. [2] used a linear mixed model fit with REML in R to quantify the contribution of variation across individuals and tissues to expression variation in GTEx.
- Burrows, et al. [3] used a linear mixed model in R to decompose the variation in expression and methylation in human induced pluripotent stem cells into the contributions of donor individual and cell type of origin.
- Rouhani, et al. [4] examined how multiple dimensions of variation affect expression variation in human induced pluripotent stem cells. This analysis uses a sophisticated custom algorithm to give a genome-wide summary of the contribution of each component. However, this method does not give gene-level results and software is not currently available.
- Trabzuni and Thomson [5] propose a linear mixed model with a finite mixture model extension to test for differential expression. However, the software requires extensive statistical experience and is not very accessible to non-experts.

Yet no user-friendly software is available. This valuable line of analysis is currently only possible for analysts with advanced knowledge of computational statistics, linear mixed models, efficient parallel computing in R, and visualization in R.

variancePartition addresses this gap in the field. By providing a computational workflow involving a few lines of R code, variancePartition allows anyone with basic proficiency in R to apply this powerful analysis framework to a range of gene expression datasets.

1.2 Interpretation of percent variance explained

In the main text, we considered a model with only one random effect. Here we extend the intuition about intra-class correlation to a model with two random effects. Consider the ith sample from the kth individual and the cth cell type:

$$y_{i,k,c} = \mu + \alpha_k^{(id)} + \alpha_c^{(cell)} + e_{i,k,c} \quad (1)$$

$$\alpha_k^{(id)} \sim \mathcal{N}(0, \sigma^2_{id}) \quad (2)$$

$$\alpha_c^{(cell)} \sim \mathcal{N}(0, \sigma^2_{cell}) \quad (3)$$

$$e_{i,k,c} \sim \mathcal{N}(0, \sigma^2_{\varepsilon}) \quad (4)$$

Applying a generalization of the simpler derivation show in the main text, two samples are correlated according to:

individual	cell type	variance	interpretation	Correlation value
same	different	$\frac{\sigma^2_{id}}{\sigma^2_{id}+\sigma^2_{cell}+\sigma^2_{\varepsilon}}$	FVE by individual	$\text{ICC}_{individual}$
different	same	$\frac{\sigma^2_{cell}}{\sigma^2_{id}+\sigma^2_{cell}+\sigma^2_{\varepsilon}}$	FVE by cell type	ICC_{cell}
same	same	$\frac{\sigma^2_{id}+\sigma^2_{cell}}{\sigma^2_{id}+\sigma^2_{cell}+\sigma^2_{\varepsilon}}$	sum of FVE by individual & cell type	$\text{ICC}_{individual,cell}$
different	different	$\frac{0}{\sigma^2_{id}+\sigma^2_{cell}+\sigma^2_{\varepsilon}}$	samples are independent	

Notice that the correlation between samples from the same individual and same cell type corresponds to the sum of the fraction explained by individual plus the fraction explained by cell type. This defines ICC for individual and tissue, as well as the combined ICC and relates these values to FVE.

In order to further illustrate how this FVE and ICC relate to the correlation between samples in multilevel datasets, consider the covariance matrix between a subset of samples from this model. Consider 5 samples from 2 individuals and 2 tissues:

Sample	Individual	Cell type
a	1	T-Cell
b	1	T-Cell
c	1	monocyte
d	2	T-Cell
e	2	monocyte

Modeling the separate effects of individual and tissue gives the following covariance structure between samples when a linear mixed model is used:

$cov(y) =$

$$
\begin{array}{c} \\ a \\ b \\ c \\ d \\ e \end{array}
\begin{array}{c}
\begin{array}{ccccc} a & b & c & d & e \end{array} \\
\begin{pmatrix}
\sigma^2_{id} + \sigma^2_{cell} + \sigma^2_{\varepsilon} & & & & \\
\sigma^2_{id} + \sigma^2_{cell} & \sigma^2_{id} + \sigma^2_{cell} + \sigma^2_{\varepsilon} & & & \\
\sigma^2_{id} & \sigma^2_{id} & \sigma^2_{id} + \sigma^2_{cell} + \sigma^2_{\varepsilon} & & \\
\sigma^2_{cell} & \sigma^2_{cell} & 0 & \sigma^2_{id} + \sigma^2_{cell} + \sigma^2_{\varepsilon} & \\
0 & 0 & 0 & \sigma^2_{id} & \sigma^2_{id} + \sigma^2_{cell} + \sigma^2_{\varepsilon}
\end{pmatrix}
\end{array}
$$

The covariance matrix is symmetric so that blank entries take the value on the opposite side of the diagonal. The covariance can be converted to correlation by dividing by $\sigma^2_{id}+\sigma^2_{cell}+\sigma^2_{\varepsilon}$, and this gives the results from above. This example generalizes to any number of variance components [6].

1.3 Variation within multiple subsets of the data

The linear mixed model underlying variancePartition allows the effect of one variable to depend on the value of another variable. Statistically, this is called a varying coefficient model [6, 7]. This model arises in variancePartition analysis when the variation explained by individual depends on tissue or cell type.

A given sample is only from one cell type, so this analysis asks a question about a subset of the data. The data is implicitly divided into subsets base on cell type and variation explained by individual is evaluated within each subset. The data isn't actually divided into subsets, but the statistical model essentially considers samples within each cell type. This subsetting means that the variance fractions no longer sum to 1.

Consider a concrete example with variation from across individual and cell types (T-cells and monocytes). Modeling the variation across individuals within cell type corresponds to the model for the ith sample, k^{th} individual, s^{th} sex and c^{th} cell type:

$$
\begin{aligned}
y_{i,k,s,c} &= \mu + \alpha^{(sex)}_s + \alpha^{(Tcell|id)}_{k,c} + \alpha^{(mono|id)}_{k,c} + e_{i,k,s,c} && (5)\\
\alpha^{(sex)}_s &\sim \mathcal{N}(0, \sigma^2_{sex}) && (6)\\
\alpha^{(Tcell|id))}_{k,c} &\sim \mathcal{N}(0, \sigma^2_{Tcell|id}) \text{ if c = Tcell, else 0} && (7)\\
\alpha^{(mono|id)}_{k,c} &\sim \mathcal{N}(0, \sigma^2_{mono|id}) \text{ if c = mono, else 0} && (8)\\
e_{i,k,s,c} &\sim \mathcal{N}(0, \sigma^2_{\varepsilon}) && (9)
\end{aligned}
$$

This model has corresponding variance components:

Variance component	Interpretation
σ^2_{sex}	variance across sex
$\sigma^2_{Tcell\|id}$	variance across individuals within T-cells
$\sigma^2_{mono\|id}$	variance across individuals within monocytes
σ^2_{ε}	residual variance

In this case the covariance matrix is no longer 'proper', because the diagonal values are not equal. But the covariance structure can still be decomposed into the contribution of each variable.

$cov(y) =$

$$
\begin{array}{c c c c c c}
 & \text{a} & \text{b} & \text{c} & \text{d} & \text{e} \\
\text{a} & \sigma^2_{Tcell|id} + \sigma^2_{sex} + \sigma^2_{\varepsilon} & & & & \\
\text{b} & \sigma^2_{Tcellid|id} + \sigma^2_{sex} & \sigma^2_{Tcell|id} + \sigma^2_{sex} + \sigma^2_{\varepsilon} & & & \\
\text{c} & \sigma^2_{sex} & \sigma^2_{sex} & \sigma^2_{mono|id} + \sigma^2_{sex} + \sigma^2_{\varepsilon} & & \\
\text{d} & \sigma^2_{Tcell|id} & \sigma^2_{Tcell|id} & 0 & \sigma^2_{Tcell|id} + \sigma^2_{sex} + \sigma^2_{\varepsilon} & \\
\text{e} & 0 & 0 & \sigma^2_{mono|id} & \sigma^2_{sex} & \sigma^2_{mono|id} + \sigma^2_{sex} + \sigma^2_{\varepsilon}
\end{array}
$$

Since the dataset is now divided into multiple subsets, direct interpretation of the fraction of variation explained (FVE) as intra-class correlation does not apply. Instead, we compute an *ad hoc* pseudo-FVE by approximating the total variance attributable to cell type by using a weighted average of the within cell type variances weighted by the sample size within each cell type. Thus the values of pseudo-FVE do not have the simple interpretation as in the standard application of variancePartition, but nonetheless allows ranking of variables based on genome-wide contribution to variance and enables analysis of gene-level results.

1.4 Software

The variancePartition package is available from Bioconductor and this manuscript coincides with variancePartition v1.0.7

Package:
`http://bioconductor.org/packages/variancePartition`

Vignette:
`https://bioconductor.org/packages/release/bioc/vignettes/variancePartition/inst/doc/variancePartition.pdf`

The vignette contains extensive documentation on the software package, general usage and visualization tools.

The software can be installed with Bioconductor's automated installer if Bioconductor v3.2 is used. Otherwise, the package can be installed from source:

```
R CMD INSTALL variancePartition_1.0.7.tar.gz
```

1.5 Simulations

Uncorrelated variables

Empirical simulations were used to evaluate the performance of the variancePartition framework. This general framework comprises 3 methods: 1) linear mixed model estimated with maximum likelihood (LMM-LM); 2) linear mixed model estimated with restricted maximum likelihood (REML); and 3) fixed effect analysis of variance (ANOVA). Continuous gene expression data was simulated according to the linear model from Equations 1-3 in the main text. All simulations used a combination of discrete and continuous variables to mimic complex study designs. The number of categories for discrete variables ranged from 2 to 100. For each sample, the value of each variable was assigned randomly so that the variables are statistically independent. Each simulation was run at two sample sizes: A) 200 and B) 1000. Each simulation included 1000 simulated gene expression traits where the true variance fractions were drawn randomly. The 3 simulation conditions are defined as follows:

Simulation 1:
variable 1: 5 categories
variable 2: 2 categories
variable 3: continuous

Simulation 2:
variable 1: 100 categories
variable 2: 20 categories
variable 3: 5 categories
variable 4: 2 categories
variable 5: continuous

Simulation 3:
variable 1: 100 categories
variable 2: 20 categories
variable 3: 20 categories
variable 4: 10 categories
variable 5: 5 categories

variable 6: 2 categories
variable 7: continuous

The variance fractions were estimated with all methods and estimates were compared to the true variance fractions to evaluate performance of the competing statistical methods (Supplementary Figure 1). The overall performance of each method was evaluated under each simulation condition using the root mean squared error (rMSE) across all variance fraction estimates (Supplementary Figure 2).

The linear mixed model estimates with maximum likelihood (LMM-LM) performed well under all conditions and demonstrated only small deviations from the true parameter values even at finite samples sizes. LMM-LM shows the smallest rMSE in all but simulation 1B, where it is very competitive. This good overall performance is likely due to the fact that LMM-LM integrates out the parameter values for each category so that it only estimates $k+1$ parameters (i.e. k variance components, plus the residuals) when k variables are included in the model [6, 8]. The linear mixed model improves performance by borrowing information across the multiple categories within the same variable. The benefit of this regularization is most evident for variables with many categories. Also notable is that fact that the accuracy and precision of the estimates are not very dependent on the number of categories in a variable for the LMM-LM method.

Conversely, restricted maximum likelihood (REML) and fixed effects ANOVA methods performed very poorly under some conditions. REML is designed to produce unbiased estimates of individual variance components [9], but it appears to perform poorly when estimating ratios of variance components. REML shows substantial deviation from the true parameter values and is inferior to LMM-LM in all simulations. REML also shows similar deviation from the true parameter values when only random effects are used (results not shown).

The fixed effects ANOVA performs well when variables contain few categories (i.e. simulation 1), but this method performs very poorly in simulations 2 and 3 where variables have up to 100 categories. This is due to the fact that the fixed effects ANOVA must estimate a regression coefficient for each category in each variable and then use these values to construct variance component estimates [7, 8]. As the number of parameters approaches the number of samples, this approach performs poorly since the model overfits the data.

Nested variables

Complex study designs often involve correlated variables and especially variables that are nested within another variable. 'Nesting' [6] refers to a situation that arises when considering variation across individuals and any properties of these individuals that do not vary in the dataset. For example, sex, age, ancestry and BMI are static properties of each individual and do not vary within individual (assuming the study is not longitudinal). The problem with nested variables is that the BMI of each individual is constant so that BMI can be

constructed as a linear combination of individual. Therefore, a fixed effects ANOVA model cannot consider both variation across individual and BMI because the model is singular and degenerate.

Yet the linear mixed model can accurately decompose variance even in cases where ANOVA is not applicable. The regularization used by the linear mixed model allows it to estimate the contribution of variables even when variables are correlated or nested. Repeating the previous simulation study except generating the continuous variable so that it is nested within the first variable (i.e. like BMI) indicates that the linear mixed model estimated with maximum likelihood produces unbiased estimates of the variance fractions under all conditions (Supplementary Figure 3). The rMSE from the maximum likelihood estimates is slightly smaller than from REML (Supplementary Figure 4). We note that the fixed effects ANOVA could not be fit on these data because the nested variables make the model degenerate.

1.6 Processing expression data

GEUVADIS

RNA-seq data was downloaded from EBI (`http://www.geuvadis.org/web/geuvadis/RNAseq-project`) and processed with limma/voom [10]. The variables Individual, Lab, Ancestry and Sex were modeled as random effects since they are discrete.

GTEx

RNA-seq data (phs000424.v4.p1) (i.e. version 4) downloaded from dbGAP and GTEx website (`http://www.gtexportal.org/`) and processed with limma/voom [10]. Only RIN was modeled as a continuous fixed effect. All other variables were modeled as discrete random effects as they are coded in the GTEx metadata: Age is discretized into 10 year bins and Ischemic time is discretized according to Supplementary Table 2.

Analysis included 489 RNA-seq experiments for 4 tissues (blood, blood vessel, skin, adipose) according to the SMTS variable. In some cases, these samples represented multiple tissue subtypes (SMTSD). Note that SMTS and SMTSD are the sample descriptors used by the .

Tissue type	Tissue subtype
Adipose	Subcutaneous, Visceral (Omentum)
Blood	Whole Blood
Blood vessel	Aorta, Coronary, Tibial
Skin	Not Sun Exposed (Suprapubic), Sun Exposed (Lower leg)

ImmVar

Expression array data was downloaded from GEO (GSE56035) and normalized with RMA [11] in the *oligo* Bioconductor package [12]. Analysis included experiments from individuals

observed in both cell types. Age was modeled as a fixed effect because it is continuous. All other variables were modeled as random effects.

SEQC

RNA-seq data was downloaded from GEO (GSE47774) and processed with limma/voom [10]. All varibles are discrete and were modeled as random effects.

2 Supplementary Figures

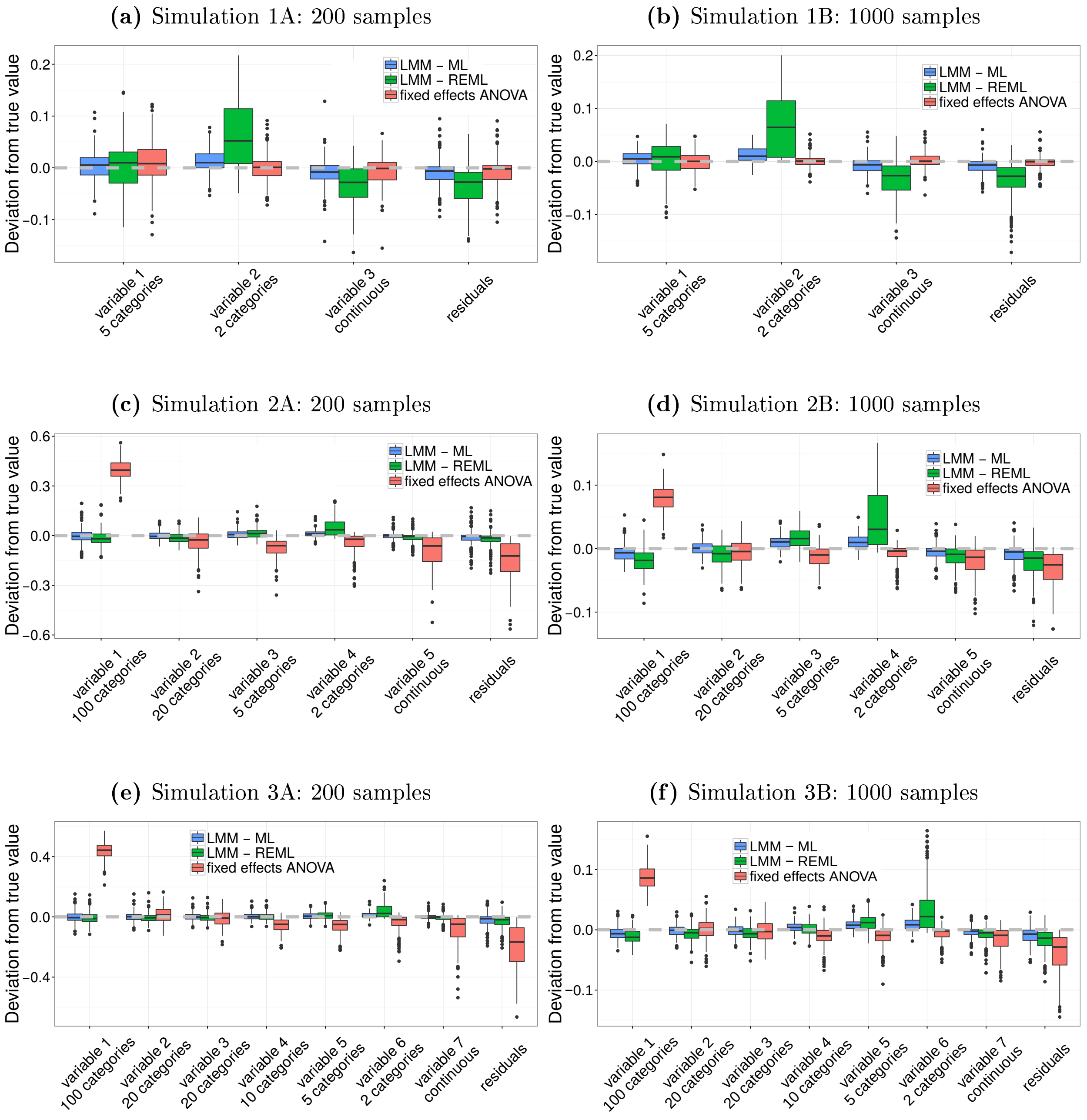

Supplementary Figure 1: Deviation of variance fraction estimates from true value for 3 statistical methods, 3 simulation conditions and 2 sample sizes. Dashed line indicates zero deviation from the true variance fraction, and boxplots indicate variation around this value for each variable and residuals for 1000 gene expression traits. **a)** Simulation 1A. **b)** Simulation 1B. **c)** Simulation 2A. **d)** Simulation 2B. **e)** Simulation 3A. **f)** Simulation 3B.

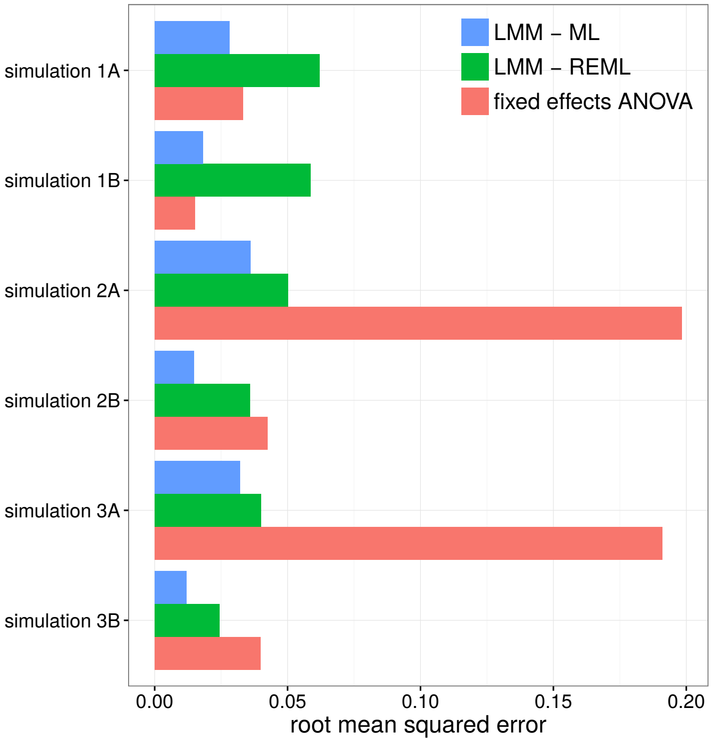

Supplementary Figure 2: Root mean squared error (rMSE) of all variance fraction estimates for each simulation condition and each statistical method. rMSE was calculated by combining the estimates from all variables and the residuals into a single value to summarize the performance of each method.

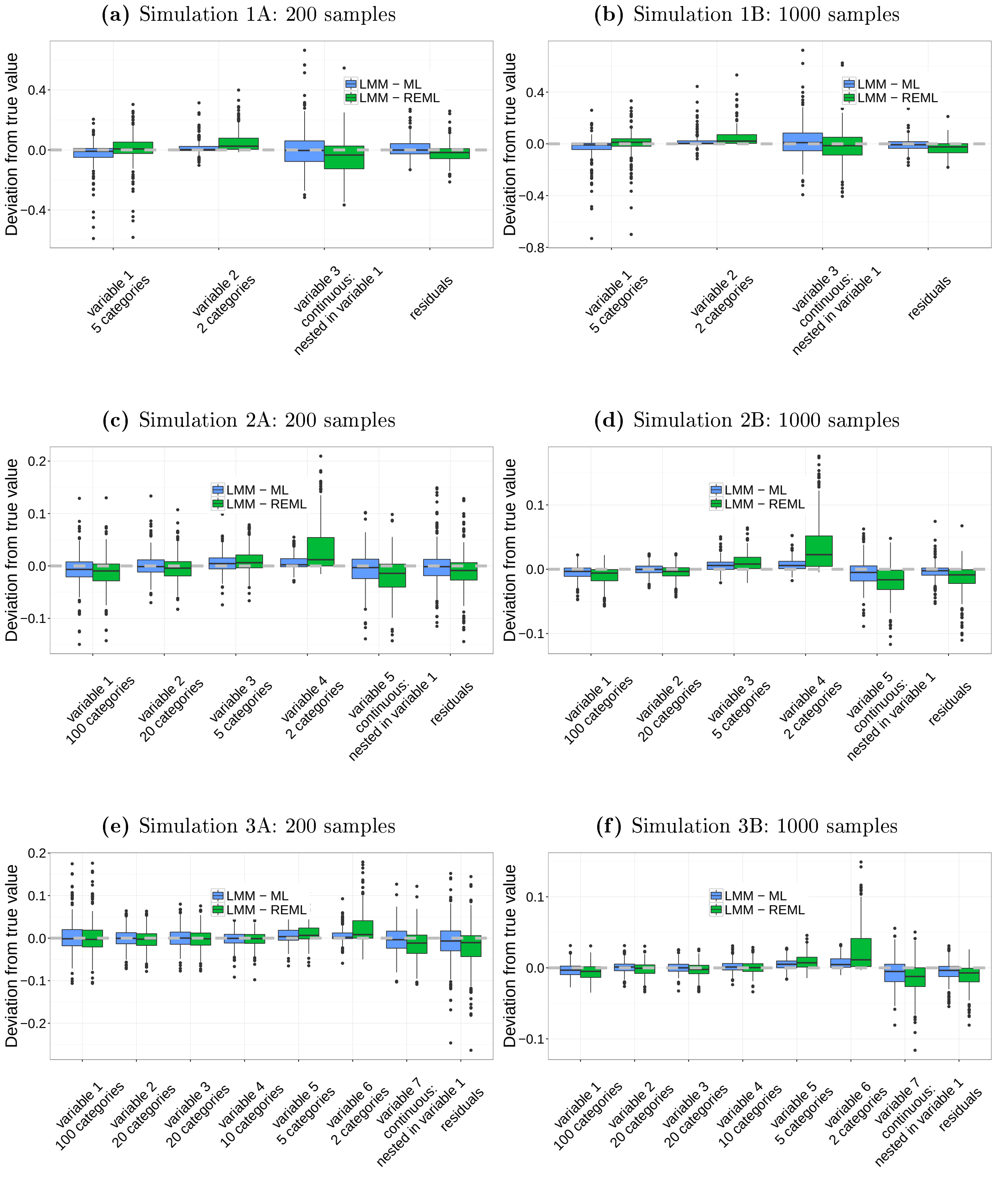

Supplementary Figure 3: Deviation of variance fraction estimates from true value for 2 statistical methods, 3 simulation conditions and 2 sample sizes where one variable is nested with another. Dashed line indicates zero deviation from the true variance fraction, and boxplots indicate variation around this value for each variable and residuals for 1000 gene expression traits. **a)** Simulation 1A. **b)** Simulation 1B. **c)** Simulation 2A. **d)** Simulation 2B. **e)** Simulation 3A. **f)** Simulation 3B.

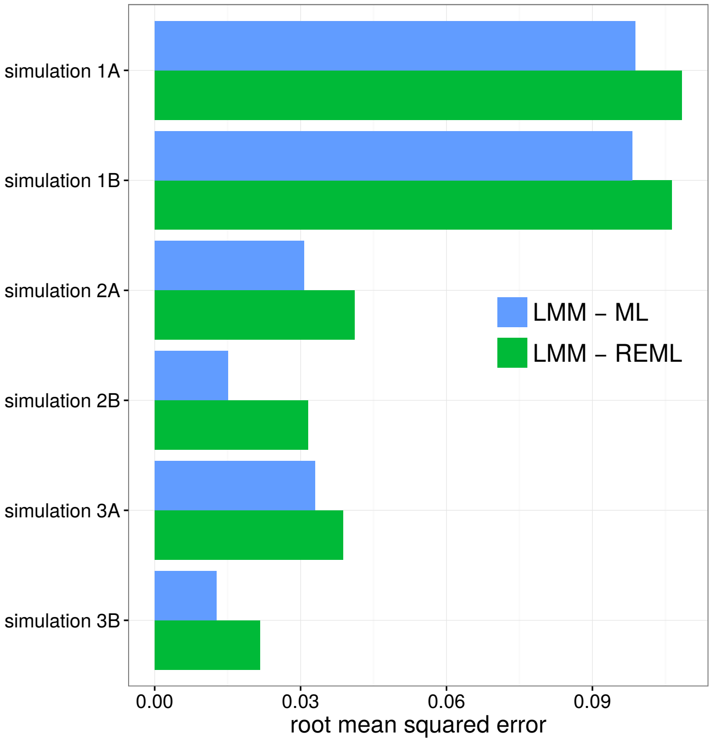

Supplementary Figure 4: Root mean squared error (rMSE) of all variance fraction estimates for each simulation condition and each statistical method. rMSE was calculated by combining the estimates from all variables and the residuals into a single value to summarize the performance of each method.

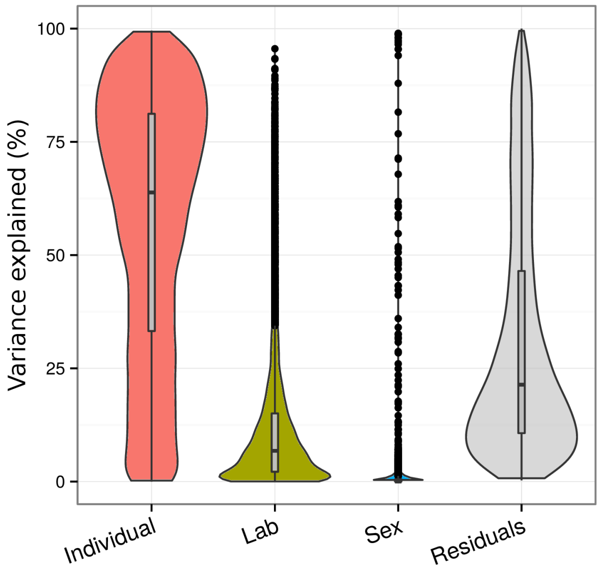

Supplementary Figure 5: Analysis of GEUVADIS dataset removing the population variable. Removing the effect of population from the analysis increased the median fraction of variation explained by variation across individual to 63.8%. Since population is necessarily a property of each individual, including population in the model will reduce the variance explained by individual. By removing population from the model, the fraction of variation explained by individual increases.

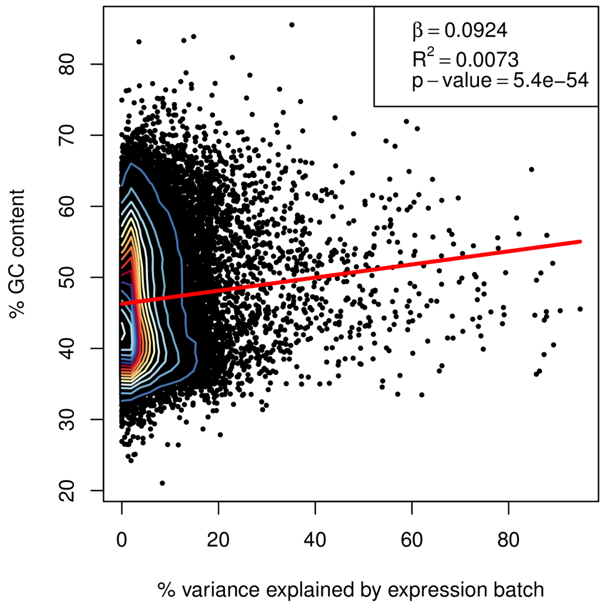

Supplementary Figure 6: Relationship between batch effect and GC content in GTEx. Scatter plot of percent GC content and percent variance explained by expression batch. Red line indicates linear regression line with regression coefficient, coefficient of determination and p-value shown.

3 Supplementary Tables

Subtissue	Adipose Tissue	Blood	Blood Vessel	Skin
Adipose - Subcutaneous	98	0	0	0
Adipose - Visceral (Omentum)	23	0	0	0
Artery - Aorta	0	0	54	0
Artery - Coronary	0	0	26	0
Artery - Tibial	0	0	89	0
Skin - Not Sun Exposed (Suprapubic)	0	0	0	22
Skin - Sun Exposed (Lower leg)	0	0	0	74
Whole Blood	0	103	0	0

Supplementary Table 1: Tissue of origin details for the 489 samples from analysis of GTEx data for 4 tissues and 8 subtissues.

Ischemic time	Adipose Tissue	Blood	Blood Vessel	Skin
-16-(-19) hours	0	1	0	0
-20-(-23) hours	0	1	0	0
-4-(-7) hours	0	2	0	0
0-(-3) hours	0	52	0	0
0-3 hours	51	9	74	38
12-15 hours	4	6	5	3
16-19 hours	6	5	7	6
20-23 hours	4	3	6	4
4-7 hours	41	15	55	35
8-11 hours	15	9	22	10

Supplementary Table 2: Ischemic time of 489 samples from analysis of GTEx data. Negative time indicates sample was taken before death.

References

[1] 't Hoen, P.a.C., Friedländer, M.R., Almlöf, J., Sammeth, M., Pulyakhina, I., Anvar, S.Y., Laros, J.F.J., Buermans, H.P.J., Karlberg, O., Brännvall, M., den Dunnen, J.T., van Ommen, G.-J.B., Gut, I.G., Guigó, R., Estivill, X., Syvänen, A.-C., Dermitzakis, E.T., Lappalainen, T.: Reproducibility of high-throughput mRNA and small RNA sequencing across laboratories. Nature Biotechnology **31**(11), 1015–22 (2013)

[2] Mele, M., Ferreira, P.G., Reverter, F., DeLuca, D.S., Monlong, J., Sammeth, M., Young, T.R., Goldmann, J.M., Pervouchine, D.D., Sullivan, T.J., Johnson, R., Segre, A.V., Djebali, S., Niarchou, A., Consortium, T.G., Wright, F.a., Lappalainen, T., Calvo, M., Getz, G., Dermitzakis, E.T., Ardlie, K.G., Guigo, R.: The human transcriptome across tissues and individuals. Science **348**(6235), 660–665 (2015)

[3] Burrows, C.K., Banovich, N.E., Pavlovic, B.J., Patterson, K., Gallego Romero, I., Pritchard, J.K., Gilad, Y.: Genetic Variation, Not Cell Type of Origin, Underlies the Majority of Identifiable Regulatory Differences in iPSCs. PLoS Genetics **12**(1), 1005793 (2016)

[4] Rouhani, F., Kumasaka, N., de Brito, M.C., Bradley, A., Vallier, L., Gaffney, D.: Genetic Background Drives Transcriptional Variation in Human Induced Pluripotent Stem Cells. PLoS Genetics **10**(6), 1004432 (2014)

[5] Trabzuni, D., Thomson, P.C.: Analysis of gene expression data using a linear mixed model/finite mixture model approach: Application to regional differences in the human brain. Bioinformatics **30**(11), 1555–1561 (2014)

[6] Pinheiro, J., Bates, D.: Mixed-Effects Models in S and S-PLUS. Springer, New York (2000)

[7] Galecki, A., Burzykowski, T.: Linear Mixed Effects Modeling Using R. Springer, New York (2013)

[8] Sorensen, D., Gianola, D.: Likelihood, Bayesian and MCMC Methods in Quantitative Genetics. Springer, New York (2002)

[9] Harville, D.A.: Maximum likelihood approaches to variance component estimation and to related problems. Journal of the American Statistical Association **72**(358), 320–338 (1977)

[10] Law, C.W., Chen, Y., Shi, W., Smyth, G.K.: Voom: precision weights unlock linear model analysis tools for RNA-seq read counts. Genome Biology **15**(2), 29 (2014)

[11] Irizarry, R.A., Bolstad, B.M., Collin, F., Cope, L.M., Hobbs, B., Speed, T.P.: Summaries of Affymetrix GeneChip probe level data. Nucleic Acids Research **31**(4), 15 (2003)

[12] Carvalho, B.S., Irizarry, R.A.: A framework for oligonucleotide microarray preprocessing. Bioinformatics **26**(19), 2363–2367 (2010)
